# Development and Characterization of a 3D Printed Cocoa Bean Shell Filled Recycled Polypropylene for Sustainable Composites

**DOI:** 10.3390/polym13183162

**Published:** 2021-09-18

**Authors:** Maria A. Morales, Alejandro Maranon, Camilo Hernandez, Alicia Porras

**Affiliations:** 1 Grupo de Diseño de Productos y Procesos (GDPP), Department of Chemical and Food Engineering, Universidad de los Andes, CR 1 18a 12, Bogotá 111711, Colombia; ma.morales12@uniandes.edu.co; 2 Structural Integrity Research Group, Department of Mechanical Engineering, Universidad de los Andes, CR 1 18a 12, Bogotá 111711, Colombia; emaranon@uniandes.edu.co; 3 Sustainable Design in Mechanical Engineering Research Group (DSIM), Department of Mechanical, Engineering, Escuela Colombiana de Ingenieria Julio Graravito, Autopista Norte AK 45 205 59, Bogotá 111166, Colombia; camilo.hernandez@escuelaing.edu.co

**Keywords:** natural filled composites, 3D printing, fused filament fabrication, cocoa bean shell, recycled polypropylene

## Abstract

Natural filler-based composites are an environmentally friendly and potentially sustainable alternative to synthetic or plastic counterparts. Recycling polymers and using agro-industrial wastes are measures that help to achieve a circular economy. Thus, this work presents the development and characterization of a 3D printing filament based on recycled polypropylene and cocoa bean shells, which has not been explored yet. The obtained composites were thermally and physically characterized. In addition, the warping effect, mechanical, and morphological analyses were performed on 3D printed specimens. Thermal analysis exhibited decreased thermal stability when cacao bean shell (CBS) particles were added due to their lignocellulosic content. A reduction in both melting enthalpy and crystallinity percentage was identified. This is caused by the increase in the amorphous structures present in the hemicellulose and lignin of the CBS. Mechanical tests showed high dependence of the mechanical properties on the 3D printing raster angle. Tensile strength increased when a raster angle of 0° was used, compared to specimens printed at 90°, due to the load direction. Tensile strength and fracture strain were improved with CBS addition in specimens printed at 90°, and better bonding between adjacent layers was achieved. Electron microscope images identified particle fracture, filler-matrix debonding, and matrix breakage as the central failure mechanisms. These failure mechanisms are attributed to the poor interfacial bonding between the CBS particles and the matrix, which reduced the tensile properties of specimens printed at 0°. On the other hand, the printing process showed that cocoa bean shell particles reduced by 67% the characteristic warping effect of recycled polypropylene during 3D printing, which is advantageous for 3D printing applications of the rPP. Thereby, potential sustainable natural filler composite filaments for 3D printing applications with low density and low cost can be developed, adding value to agro-industrial and plastic wastes.

## 1. Introduction

Over the last decades, global environmental issues have become noticeable, including increased energy consumption, shortage of petrochemical resources, greenhouse gas exhaustion, and accumulation of plastic waste [1,2]. These environmental concerns and economic factors have motivated researchers to investigate environmentally friendly materials with sustainability benefits [3,4,5], where one of the benefits is the potential to move towards a circular economy [6].

Polymeric materials are widely used daily, thanks to their unique properties [7]. Consequently, plastic accumulation in the natural environment and landfills causes adverse environmental impacts. In the early 2000s, about 65–70% of plastic products were finally disposed of in landfills, 20–25% were incinerated, and just 10% were recycled [8], causing an ocean pollution rate of about 12.7 million tons of plastic per year [9], even making evident the presence of plastics in the food chain [10,11]. On the other hand, agricultural-based industries produce high amounts of residues, causing pollution and harmful effects on humans and animals’ health [12]. Specifically, in Colombia, cocoa bean production reached 63,416 tons in 2020 [13]. Pod husk, pulp, and bean shell, representing about 80 wt.% of cocoa fruit, are considered waste and are left on cacao crops, causing environmental problems [14,15]. Accordingly, the cocoa industry has been trying to find added-value applications to these by-products as bio-recyclable paper packing [16], fertilizers [17], human and veterinary supplements [18], among others [17].

With the challenge of reducing plastic usage and correctly disposing of agro-industrial wastes, natural composites based on recycled or biodegradable polymers are receiving more attention due to their ecologically friendly behavior, flexibility, low cost, low density, and ease of fabrication [19,20,21]. From this perspective, vegetable origin fibers as wood flour, rice husk, coconut husk, hemp, and flax have been widely used as fillers in composite materials [22,23,24]. However, few studies have implemented cocoa bean shells (CBS) in this field. Puglia et al. studied tensile, thermal, and morphological properties of polycaprolactone (PCL)/CBS composites by injection molding system [25]. Papadopoulou et al. worked on sustainable active food packaging based on CBS/polylactic acid (PLA) by dissolution PLA in chloroform [26]. Tran et al. developed biofilaments based on PCL/CBS for 3D printing applications [27]. Altogether, a number of documented studies on cocoa production and by-product generation have assembled to create new material development opportunities with promising possibilities working with 3D printing technology and recycled industrial polymers such as polypropylene, one of the most common polymers. Just in the 2020, the global polypropylene production had a capacity of 88.6 million MT.

Likewise, new advanced technologies, as 3D printing, allow updating current manufacturing activities for more sustainable ones [28,29]. Traditional manufacturing technologies can be wasteful, consuming large amounts of raw materials. Rather than sculpting an item from a piece of plastic, by 3D printing the item is manufactured layer-by-layer. There is less waste, between 70% and 90%, than traditional methods such as injection molding [30]. Additionally, 3D printing contributes to sustainable design thanks to its flexibility in manufacturing materials and customization capacity. Fused Filament Fabrication (FFF) develops materials through layer-by-layer addition using 3D model data [31,32]. This 3D printing process allows complex shapes with reduced material waste and manufacturing time [3,33,34]. Materials used in the FFF technology are filaments with a specific diameter [35]. Filaments are fed into a nozzle, where the material is melted by heating above glass transition or melting temperature [36]. Following a computer-assisted design (CAD) model, the nozzle moves and deposits the melted material layer-by-layer [31,37,38]. For the FFF process, amorphous polymers are preferred over semicrystalline ones because amorphous polymers have lower solidification shrinkage and a liquid-like structure in the solid-state [39]. In particular, 3D printing with polypropylene is a complex process. It induces rapid shrinkage and warping [40,41], which causes the part to become curved and unsticks from the printing platform [33], making it challenging to 3D print. According to Stoof et al. and Milosevic et al., reinforcing semicrystalline matrixes with natural fillers may reduce the shrinkage and warping effect [40]. However, few natural fibers have been studied in 3D printing with polypropylene [41].

Currently, the comprehensive 3D printing market is growing. There is an interest to recycle plastic and agricultural waste into a standardized filament product for the 3D printing industry because it gives a possibility of valorization—a second life—and enables effective waste utilization to obtain consumable products [36,40]. However, the use of recycled polypropylene filled with cocoa bean shells remains unexplored. Hence, this study presents the development and characterization of a 3D printing composite filament based on recycled polypropylene and cocoa bean shells. Density, water absorption, swelling diameter, thermogravimetric analysis (TGA), differential scanning calorimetry (DSC), warping analysis, tensile test, and scanning electron microscopy (SEM) were performed to evaluate 3D printed specimens’ properties using 5 wt.% of particulate CBS. Furthermore, to evaluate the dependence of mechanical behavior on the printing direction, two different raster angles were chosen to print the specimens.

## 2. Materials and Methods

### 2.1. Materials

Promaplast S.A.S supplied homogenized recycled polypropylene pellets recovered from post-industry use (rPP), with a melt flow rate of 5.11 g/10 min. Casaluker S.A. provided cocoa bean shells (CBS) were collected from Necocli from Antioquia Department in Colombia, with a density of 0.41 ± 0.04 g/cm^3^ [42].

### 2.2. Processing of Composite Filaments

**Figure 1:**
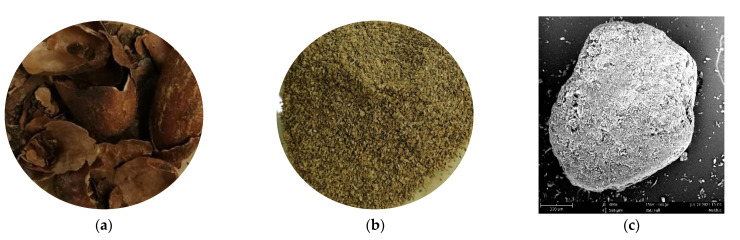
(**a**) CBS, (**b**) particulate CBS, and (**c**) CBS particle.

Composite filaments of rPP/CBS with 5 wt.% of CBS were produced using the following steps. First, the CBS was ground using a Pulverisette 19 mill to reduce the particle’s size and sieved on No. 40 and No. 60 sieves (ASTM E11 [43]), obtaining a particle filler size range between 250 and 425 µm (Figure 1).

Second, particulate CBS and rPP pellets were dried at 105 °C for three hours before extrusion to prevent voids formation in the final feedstock.

**Figure 2:**
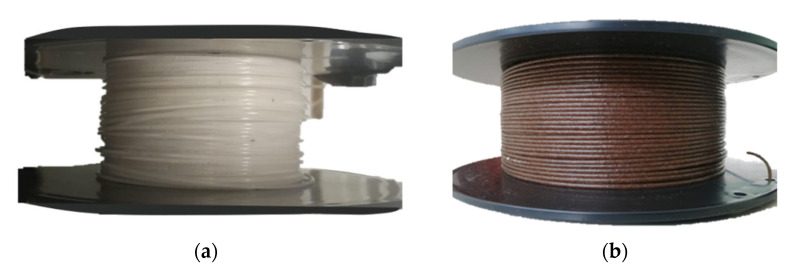
(**a**) rPP and (**b**) rPP/CBS 5 wt.% 3D printing filaments.

Third, the extrusion of the materials was made on a Brabender DSE 20 twin extruder with six temperature-controlled zones. A temperature profile between 175 to 190 °C was used. The screw speed was maintained between 6 and 13 rpm. The obtained filament was cooled in water at room temperature and granulated in a pelletizer. A second extrusion step was performed to improve the homogeneity of the mixture, with an equal set of parameters than the first cycle. Finally, the diameter of the resulting filament was 1.75 ± 0.1 mm accomplished with a 2 mm diameter cylindrical nozzle. To compare the properties of the rPP/CBS composite filament with the neat recycled polymer, rPP filament was also produced, as shown in Figure 2.

### 2.3. Row and Feedstock Material Thermal Characterization

#### 2.3.1. Thermogravimetric Analysis (TGA)

Neat rPP, CBS particles, and rPP/CBS were thermally characterized using a thermogravimetric analyzer (SDT Q600, TA Instruments, New Castle, TE, USA). Tests were performed according to the ASTM E1131 standard using a sample weight of 2 mg. Samples were heated from room temperature to 600 °C at a 10 °C/min rate under a continuous flux of nitrogen (100 mL/min). Three samples of each material were evaluated.

#### 2.3.2. Differential Scanning Calorimetry (DSC)

Differential scanning calorimetry was used to evaluate thermal properties following the ASTM D3418 standard in a DSC Q2000 (TA Instruments, New Castle, TE, USA). Three samples of each material were heated at 10 °C/min from room temperature to 220 °C; afterward, the temperature was held for 5 min to eliminate thermal history and residual moisture. Then, the sample was cooled down to room temperature at 10 °C/min and finally reheated to 220 °C at 10 °C/min under a nitrogen atmosphere at a flow of 300 mL/min. Equation (1) was used to calculate the degree of crystallinity of the samples.

(1) $$\%\;\mathrm{crystallinity}=\left(∆H_{f}^{\mathrm{obs}}/∆H_{f}^{0}\right)/\left(1-w_{f}\right)\times 100$$

where $∆H_{f}^{\mathrm{obs}}$ is the observed enthalpy of fusion, $∆H_{f}^{0}$ is the enthalpy of fusion of the completely crystalline materials at the equilibrium melting temperature T_m_ (207 J/g [44,45]), and w_f_ is the weight fraction of the filler.

### 2.4. Feedstock Material Density

The densities of neat rPP and rPP/CBS composites were determined according to the ASTM D792 standard. Test method B was used to measure the density of the specimens using ethanol (ρ  = 0.789 g/m^3^) at 19.9 °C as the immersion liquid. Three specimens of each material were tested.

### 2.5. Row and Feedstock Material Water Absorption and Diameter Swelling

Water absorption and diameter swelling were measured using ASTM D570 standard. For water absorption and diameter swelling measurements, specimens were dried at 50 °C for 24 h, then cooled in a desiccator, and immediately weighed (W_0_). Afterward, specimens were immersed in distilled water for 2 h, and all surface water was wiped off with a dry cloth and finally weighed (W_i_). Equation (2) was used to calculate the percentage (weight) of increase during the immersion. Three specimens were evaluated by test.

Increase in weight (weight %) = (W_i_ − W_0_)/W_0_ × 100 (2)

The swelling diameter was determined using Equation (3), where D_0_ and D_i_ are the diameter of each specimen before and after the water immersion test, respectively.

Diameter swelling (%) = (D_i_ − D_0_)/D_0_ × 100 (3)

### 2.6. 3D Printing

**Figure 3:**
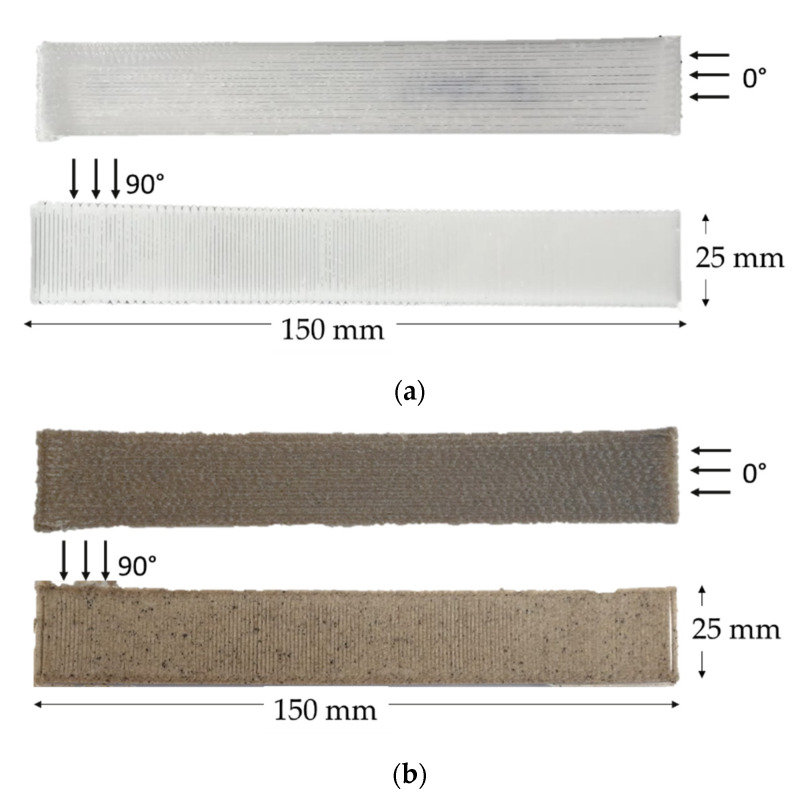
(**a**) rPP and (**b**) rPP/CBS 5 wt.% tensile specimens (150 mm × 25 mm × 2.5 mm), 3D printed at 0 and 90°.

A 3D FF-STD Doppia machine was used to print rPP and rPP/CBS filaments as tensile test specimens following the ASTM D3039 standard (Figure 3). Specimens were held fixed on the bed using a Magigoo 3D Printing Adhesive for PPGF, and a brim platform due to the warping effect. Simplify 3D software (Version 4.0.1, Simplify 3D, Cincinnati, OH, USA)was used to edit the STL file. A 90 °C bed temperature was set for the first layer, 70 °C for the remaining layers, and 250 °C for the nozzle. The layer height used was 0.25 mm, with a nozzle diameter of 0.8 mm, a printing speed of 60 mm/s, and 100% infill.

As the raster angle affects the forming accuracy and the mechanical performance of the printed samples [46], two types of specimens at different raster angles (90 and 0°) were printed using the neat rPP and rPP/RH 5 wt.% filaments to determine the tensile properties.

### 2.7. Mechanical Characterization

A universal testing machine (Instron 3367, Instron, Norwood, MA, USA), equipped with a 30 kN load cell, was used to measure the tensile properties of the specimens. According to ASTM D3039/3039M standard, the test was performed with a gauge length of 50 mm and a crosshead speed of 10 mm/min and 1.2 mm/min for specimens printed at 90° and 0°, respectively. The strain was measured for all specimens using an extensometer fixed to the samples. Young’s modulus was calculated according to the ASTM E111 standard, as the ratio of tensile stress to corresponding strain below the proportional limit, where two points on the linear section of the stress(σ)-strain(ε) curve are joined to calculate the slope of the resulting line (Equation (4)) [47]. Per composition and printing condition, five tensile specimens were tested until failure.

Young’s modulus = (σ_2_ − σ_1_)/(ε_2_ − ε_1_) (4)

### 2.8. Microcospy Analysis

To understand the failure of rPP/CBS composites printed using FFF, selected tensile tested specimens’ surfaces were studied by scanning electron microscopy (SEM). A JEOL JSM-6490LV (JEOL, Tokyo, Japan) at 10 kV was used to analyze the samples. To improve the conductivity of the specimens, they were prepared with gold-sputtering for 1 min at 20 mA.

### 2.9. Statistical Analysis

A one-way analysis of variance (ANOVA) was carried out to evaluate the filler weight ratio in-fluence in the developed materials’ physical and mechanical properties. Two levels of the filler weight ratio factor were used (0 and 5 wt.%). Density, water absorption, diameter swelling, tensile strength, fracture strain, and Young’s modulus were used as response variables. A *p*-value lower than 0.05 (confidence level of 95%) was considered statistically significant [48]. Statistical analysis was carried out for each 3D printing raster angle. Minitab 18 Statistical Software (Version 18, Minitab Inc., State College, PA, USA) was used to analyze data.

## 3. Results

### 3.1. Row and Feedstock Material Thermal Characterization

**Figure 4:**
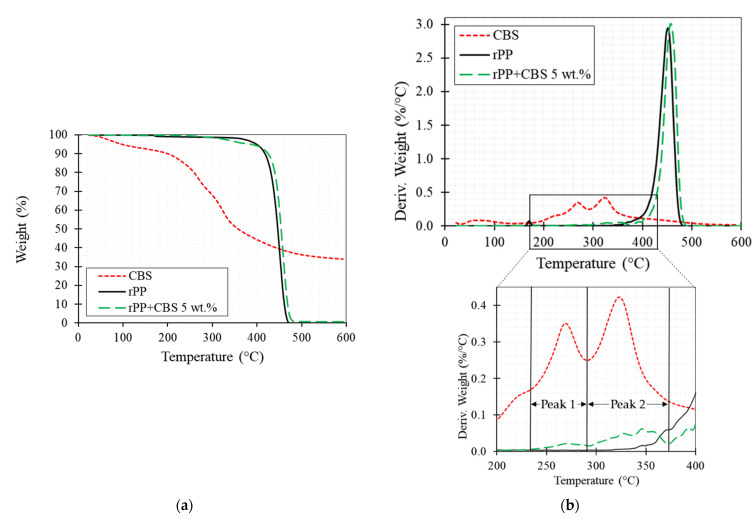
(**a**) TGA and (**b**) DTGA curves of CBS particles, rPP and rPP/CBS 5 wt.% composite filaments obtained using TGA.

The thermal stability of the neat rPP and the rPP/CBS composite 5 wt.% was investigated through TGA analysis, and the results are summarized in Figure 4. TGA analyzes the weight loss as the sample is heated at a constant rate (Figure 4a). At the same time, the DTG represents the rate of material weight changes upon heating against temperature and is used to simplify reading the weight versus temperature thermogram peaks (Figure 4b).

CBS particles show a three-phase degradation process. The first phase, up to 125 °C, with a weight loss of around 7%, is attributed to the vaporization of water in the CBS (moisture content). The second phase occurs between 230 °C and 290 °C with a weight loss of around 29%, which indicates the degradation of the hemicellulose and cellulose. The final phase, between 295 °C and 380 °C, is due to lignin and remaining cellulose degradation (weight loss around 55%). Residual char and ash content about 35% was found after 600 °C. Even though the thermogravimetric behavior of natural fillers depends on their chemical constituents [49], the thermal behavior obtained in this study coincides with the report for other lignocellulosic fibers such as flax, hemp, sugar cane, bamboo, coconut, among others [49,50,51,52].

Neat rPP shows a two-step degradation process, the first step, between 160 °C and 180 °C (with a slight weight loss around 1%), is associated with impurities present in the sample due to its recycled nature. The second step represents the main degradation process of the polymers, which occurs between 370 °C to 480 °C. The main step is caused by the cleavage of the polymer chain [27,53]. rPP/CBS composite presents a weight-loss curve that combines its constituents’ thermal behavior. The TGA curve exhibits a first weight loss of 5% between 230° and 380 °C, representing fiber degradation. In the DTGA is observed that this first phase has two main peaks, meaning the filler components degradation (hemicellulose, cellulose, and lignin). The second phase, between 390 °C and 490 °C, presents a 99% weight loss and a maximum decomposition rate at 460 °C, indicating the matrix’s decomposition. Lastly, a residual char of 1% is observed at 600 °C. Further, rPP/CBS thermal behavior is consistent with literature reported for other natural composites such as bagasse with PLA [2], Manicaria saccifera with PLA [54], flax with PLA/Thermoplastic starch (TPS) [24], sugar palm/glass fiber with polyurethane [55], and date palm fiber reinforced PP [56].

In summary, the results suggest that composite filaments must be processed below 230 °C to prevent CBS particles’ degradation. However, rPP/CBS composites are suitable for 3D printing with higher processing temperatures considering the low residence time (0.02 s per filament mm) of the material in the printer extruder.

**Figure 5:**
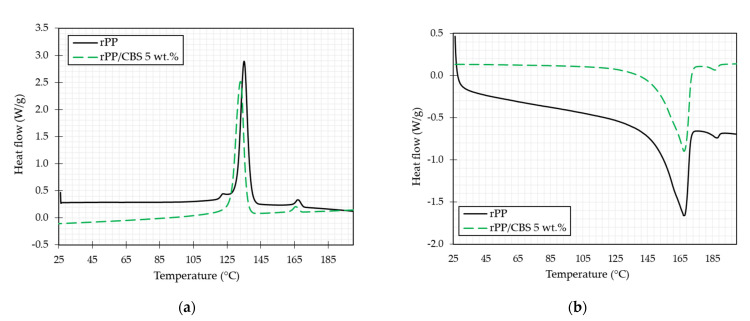
DSC (**a**) endothermal and (**b**) exothermal representative thermograms of rPP and rPP/CBS 5 wt.% composites.

DSC test measures the heat flow as a function of the temperature associated with material transitions. Exothermal and endothermal peaks represent a thermal phase transition of the samples [57]. Figure 5 illustrates endothermal (Figure 5a) and exothermal (Figure 5b) curves for rPP and rPP/CBS 5 wt.%. As shown, the addition of CBS particles to the matrix resulted in a decrease in crystallization temperature due to the hydrophobic effect of the matrix, which leads to poor interfacial properties [58]. On the other hand, melting temperature presents a slight decrease attributed to the nucleation on the CBS surface that shortened the polymer crystallization time and led to small crystals [59]. According to the thermal characterization, the suitable processing temperature for composite filaments by extrusion should be above the composite melting temperature (165 °C) and below 230 °C to promote a good particle/matrix blend and avoid the thermal degradation of the CBS.

The exotherm and endotherm curves exhibit a small peak between 160 °C and 190 °C associated with impurities present in the samples, as seen in the TGA results. The endothermal transition of this impurity occurs between 165 °C and 169 °C, while the exothermal transition occurs between 185 °C and 190 °C.

Table 1 presents the value of melting and fusion temperatures, enthalpies, and crystallinity of the samples. There is a decrease in the melting enthalpy of the composite sample. This behavior occurs because the CBS components do not suddenly melt when heated; they do not have a melting point. Similar behavior was reported by Hidalgo et al. They evaluated thermal properties of low-density polyethylene (LDPE)/aluminum (Al)/fique composites, and fique fibers do not present melt at test conditions, causing a decrease in the enthalpy of fusion when is added [60].

Crystallinity percentage tends to decrease when the CBS particles are added due to the CBS components. Hemicellulose and lignin identified in the TGA analysis are amorphous polymers, while cellulose has more crystalline regions [61,62]. This behavior coincides with results obtained by Hong et al. for PLA and Bagasse [2], and by Chatterjee et al. for PP and jute fiber [63].

### 3.2. Row and Feedstock Material Physical Properties

Physical properties of neat rPP and rPP/CBS 5 wt.% are illustrated in Table 2. Results show a decrease (1%) in the material density in composite filament compared to the neat rPP. The ANOVA analysis determines that the filler weight ratio is statistically significant (*p*-value = 0.030) for this property. This result is attributed to the density difference between the CBS (density = 0.41 ± 0.04 g/cm^3^ [42]) and the rPP matrix (0.893 g/cm^3^).

The use of natural fillers, as the cocoa bean shell, represents an opportunity for applications in industries where lightweight construction is important. For example, automotive companies use natural fiber biocomposites in the non-structural plastic parts of vehicles [64,65].

**Figure 6:**
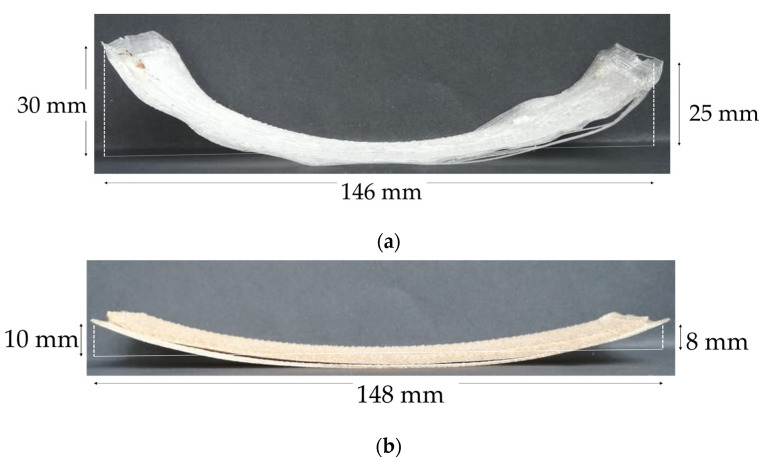
Warping behavior of 3D printed (**a**) recycled polypropylene and (**b**) rPP/CBS 5 wt.% samples.

Water absorption and diameter swelling test was performed to compare the hydrophobic behavior of neat rPP and rPP/CBS composite. Water absorption in composite materials depends on the filler moisture content, permeability, void content, and compatibility between individual components [66]. Natural fillers are hygroscopic materials, which could decrease the quality of filler-matrix bonding. Figure 6 shows that the water absorption percentage increases by 138% in the composite. According to the ANOVA test, the cocoa bean shell addition is significant on this parameter (*p*-value = 0.009). This result is in agreement with other composites studies with lignocellulosic fillers [66].

Swelling behavior tends to increase in composite material, compared to rPP, because of the greater affinity between water to the hydroxyl and oxygen groups present in the CBS [67,68,69]. However, in this case, the filler addition does not statistically significantly affect this property (*p*-value = 0.616).

The previous analysis suggests that a well-dried material is required before processing to prevent voids formation and improve the quality of 3D printed parts [39].

### 3.3. Warping Analysis

Semicrystalline thermoplastics present shrinkage during 3D printing due to tightly packed polymer chains in crystalline regions [70]. For example, Chong et al. investigated the possibility of using high-density polyethylene (HDPE) as feedstock for 3D printing. However, they found difficulties during the process due to the warpage and the adhesion of the material with the bed [71]. PP as a semicrystalline polymer also presents this behavior: Sporerk et al. even dedicated a study to review how to improve warpage behavior during 3D printing of PP [72].

In this study, during the 3D printing process of the neat rPP a warping effect was observed. As the layers solidified, shrinkage along the sample causes warping, as shown in Figure 6. With the particulate filler addition, a decrease of 67% in the warping effect was achieved. This improvement is a consequence of increasing amorphous structures in the composite compared to the neat rPP. It helps to quickly solidify with less shrinkage, which allows the printed layer to stick to the upcoming layer [73]. This result follows Pickering et al., who reported that fiber addition is an effective method to improve the warping effect in 3D printed polymers [74,75,76]. Warping and shrinkage improvement allow polypropylene application in fields like prototyping manufacturing and construction [77].

### 3.4. Mechanical Characterization

**Figure 7:**
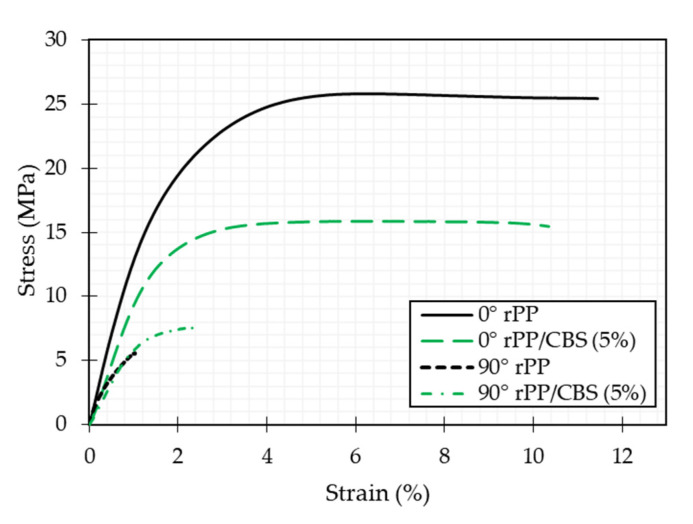
Stress-strain representative curves for rPP and rPP/CBS 5 wt.%, printed at 0 and 90°.

Figure 7 shows the representative stress-strain curves for neat rPP and rPP/CBS 5 wt.% composite printed using different raster angles (0 and 90°). These curves show an initial linear elastic region. The Young’s modulus is determined, followed by plastic deformation up to failure. Comparing mechanical behavior between both raster angles evaluated, properties at 0° are generally higher than at 90° due to the relation between the loading mechanism and the deposition direction of printed layers. In specimens printed at 0°, layers are deposited parallel to the tensile strength load.

In comparison, when specimens are printed at 90°, the layers are deposited perpendicular to the loading direction. In this way, the material’s mechanical properties are evaluated in specimens printed at 0°. In contrast, bonding between layers is considered in specimens printed at 90°. From this result, it can be concluded that tensile properties are highly dependent on the printing raster angle of the specimen.

Table 3 summarizes the mechanical properties of rPP and rPP/CBS composite. Generally, mechanical properties in composite materials are deeply dependent on factors such as filler content, dispersion in the matrix, compatibility between matrix-filler, and adhesion [78]. In specimens printed at 0°, the implementation of CBS affected the continuity of the rPP molecular chains, which causes the tensile strength, and Young’s modulus to decrease compared to rPP (*p*-value < 0.001) [70]. However, fracture strain was not affected by the filler addition (*p*-value = 0.185). A potential explanation for the loss in tensile strength of the composites, compared to the neat rPP, is the absence of chemical bonding between rPP and CBS, and the limited dispersion of the CBS in the matrix [79,80]. The obtained results are aligned with other studies where lignocellulosic fibers addition in polymeric matrixes were evaluated [81,82,83]. For example, Fuentes et al. evaluated the mechanical behavior of PP/bamboo and PP/glass fiber. They obtained poor mechanical performance for bamboo composites due to the low physical and chemical compatibility between the bamboo fiber and the matrix [84].

In specimens printed at 90°, tensile strength increases significantly by 83% for rPP/CBS 5 wt.% specimens compared to rPP (*p*-value = 0.006). This behavior could be explained by the addition of amorphous structures when the CBS particles are included. In semicrystalline materials as rPP the cooling process of each layer is quick, which means that little time is allowed to approach the equilibrium state [85], causing weak bonding between layers, shrinkage, and dimensional instability. Therefore, a decrease in crystalline structure reduces this effect and promotes a better interlayer filament composite bonding. In addition, Young’s modulus is not affected by the filler weight ratio (*p*-value = 0.834). However, the fracture strain presents a significant change (*p*-value = 0.005), indicating that the composite specimens are more ductile and have higher energy absorption capacity than neat rPP specimens when a 90° raster angle is used.

**Figure 8:**
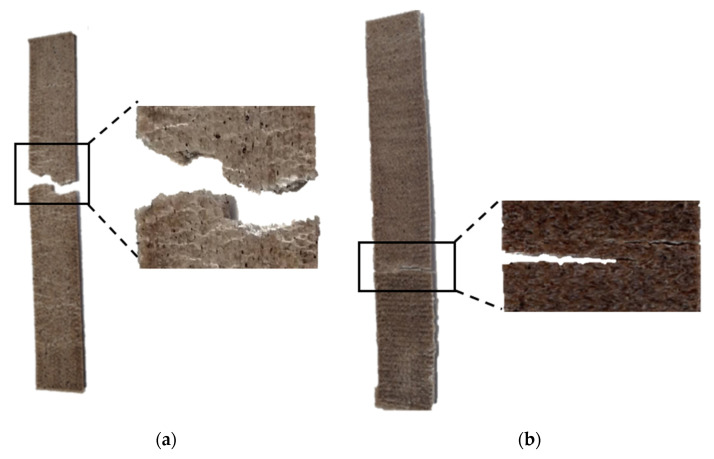
Typical tensile failure modes for rPP/CBS 5 wt.% composite printed at (**a**) 0°: Angled gage middle (AGM) failure mode, and (**b**) 90°: Lateral gage middle (LGM) failure mode.

Figure 8 shows the typical tensile failure modes for rPP/CBS 5 wt.% specimens printed at 0 and 90°. The type of failure depends on the raster angle used. According to the failure codes described in the ASTM D3039 standard, specimens printed at 0° show an angled gage middle (AGM) failure mode. An irregular fracture occurred perpendicular to the layer’s deposition direction. On the other hand, specimens printed at 90° have a lateral gage middle (LGM) failure mode. The failure occurs through the bonded layers adjacent to the layers’ deposition direction due to the limited bonding between individual printed layers [79].

### 3.5. Tensile Test Fractured Specimens Morphology

**Figure 9:**
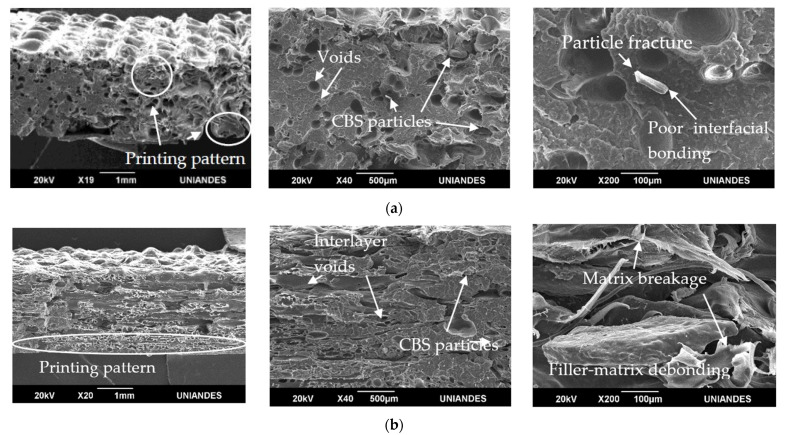
SEM images of rPP/CBS 5 wt.% tensile fractured specimens. Specimens were printed at (**a**) 0° and (**b**) at 90°. Images were acquired at different magnifications.

SEM images in Figure 9 display the fracture surface of rPP/CBS 5 wt.% tensile tested specimens. Figure 9a shows failed specimens printed at 0°. The printing pattern is perceptible due to the tubular shape that excels on the surface. Nevertheless, at higher magnifications, images show the filler-matrix poor interfacial adhesion, which produces interfacial gaps, voids formation, and non-uniform dispersion of the CBS particles in the matrix. This kind of imperfection causes stress concentrators, affecting mechanical properties. This behavior between matrix and natural filler is usually due to the inherent incompatibility of the CBS particles with hydrophobic polymer matrix and the number of functional groups (hydroxyl and other polar groups) that contribute to acid-base interaction in the contacting area [86,87]. Another aspect that affects mechanical properties when natural fillers are used is the water absorption of the filler, which can causes fibers to swell and generate micro-cracking of composites [88]. At higher magnification, particle fracture, filler-matrix debonding, and matrix breakage were observed as the central failure mechanism [89]. Numerous techniques have been studied to modify the fiber’s surface to reduce water absorption and improve bonding adhesion with the matrix [90]. One of the most used techniques is the alkali treatment, which enhances the filler-matrix compatibility by removing hemicellulose and other impurities of the particles, reducing the filler-matrix debonding [56,91,92].

Figure 9b shows specimens printed at 90°, in minor magnifications 3D printing pattern can be seen as thin sheets. However, different layers are not easy to identify, which suggests a good interlayer bonding between layers. Notably, matrix breakage and filler-matrix debonding were the principal failure mechanisms.

## 4. Conclusions

This study presented the development of a 3D printing composite filament and its characterization in thermal, physical, mechanical, and morphological properties, showing the potential to use the cocoa bean shell (CBS) and recycled polypropylene (rPP) as feedstock to produce 3D printed composite materials.

In rPP/CBS 5 wt.%, thermal degradation starts at 230 °C due to the presence of lignocellulosic materials. Even though this result suggests a processing temperature below 230 °C to avoid CBS degradation, the printing temperature could be higher due to the short residence time. The amorphous structures of the hemicellulose and cellulose, present in the CBS, induce a decrease in composite crystallinity.

The composite density decreases due to the low density of the CBS filler. Swelling diameter does not exhibit a statistically significant difference with filler addition, while water absorption increases because natural fillers have a hydrophilic behavior.

It was found that adding cocoa bean shells to recycled polypropylene during the manufacturing of filaments reduced both shrinkage and warping effect, contributing to the dimensional stability of final printed samples.

Mechanical properties showed high dependency on the raster angle, obtaining better properties when 0° was used. The load direction explains this mechanical behavior compared to the layer deposition direction. When specimens were printed at 0°, the load occurred parallel to the layers, evaluating material properties itself. In comparison, when 90° is used, the load occurred perpendicular to the layer’s deposition direction, evaluating the bonding between adjacent layers. In specimens printed at 0°, CBS acts as a filler, tensile strength, and Young’s modulus decrease when the CBS is implemented. However, in specimens printed at 90°, the CBS helps to improve adjacent layers bonding, tensile strength, and tensile elongation exhibit an increase of 83% and 5%, respectively.

SEM analysis showed particle fracture, filler-matrix debonding, and matrix breakage as the main composite failure mechanisms. These mechanisms could be caused by the poor interfacial bonding between the hydrophobic matrix and the hydrophilic CBS.

Finally, this study shows that it is possible to produce 3D printable composites filaments based on agro-industrial and polymeric wastes, such as cocoa bean shell (CBS) and recycled polypropylene (rPP), contributing to a circular economy.

## Figures and Tables

**Table 1:** Crystallization and fusion properties of rPP and rPP/CBS 5 wt.%.

Sample	Crystallization		Melting		Crystallinity (%)
	T_c_ (°C)	∆Hc (J/g)	T_m_ (°C)	∆Hm (J/g)	∆H_PP_^0^
rPP	134 ± 1	91 ± 5	167 ± 1	94 ± 3	49 ± 7
rPP/CBS 5 wt.%	134 ± 4	95 ± 5	167 ± 1	85 ± 5	43 ± 3

Values are given as mean ± standard deviation.

**Table 2:** Physical properties of rPP and rPP/CBS 5 wt.% composite.

Sample	Density (g/cm^3^)	Water Absorption (%)	Swelling Diameter (%)
rPP [12]	0.89 ± 0.01	0.29 ± 0.15	0.54 ± 0.13
rPP/CBS 5 wt.%	0.88 ± 0.01	0.93 ±0.11	0.68 ± 0.33

Values are given as mean ± standard deviation.

**Table 3:** Mechanical properties of rPP and rPP/CBS 5 wt.% composite at different raster angles.

Sample		Tensile Strength (MPa)	Fracture Strain (%)	Young’s Modulus (GPa)
0°	rPP	26.02 ± 0.47	6.16 ± 0.19	1.34 ± 0.05
	rPP/CBS 5 wt.%	15.23 ± 0.91	5.73 ± 0.63	0.95 ± 0.04
90°	rPP	4.33 ± 1.73	1.01 ± 0.35	0.74 ± 0.37
	rPP/CBS 5 wt.%	7.93 ± 1.29	2.20 ± 0.60	0.78 ± 0.14

Values are given as mean ± standard deviation.

## Data Availability

The data are available from the corresponding author upon request.
